# Cryobiopsy: A Breakthrough Strategy for Clinical Utilization of Lung Cancer Organoids

**DOI:** 10.3390/cells12141854

**Published:** 2023-07-14

**Authors:** Dongil Park, Dahye Lee, Yoonjoo Kim, Yeonhee Park, Yeon-Jae Lee, Jeong Eun Lee, Min-Kyung Yeo, Min-Woong Kang, Yooyoung Chong, Sung Joon Han, Jinwook Choi, Jong-Eun Park, Yongjun Koh, Jaehyeok Lee, YongKeun Park, Ryul Kim, Jeong Seok Lee, Jimin Choi, Sang-Hyun Lee, Bosung Ku, Da Hyun Kang, Chaeuk Chung

**Affiliations:** 1 Division of Pulmonology and Critical Care Medicine, Department of Internal Medicine, College of Medicine, Chungnam National University, Daejeon 34134, Republic of Korea; rahm3s@gmail.com (D.P.); ziczi02@naver.com (D.L.); misspims@naver.com (Y.K.); jelee0210@cnu.ac.kr (J.E.L.); ibelieveu113@naver.com (D.H.K.); 2 Division of Pulmonary and Critical Care Medicine, Department of Internal Medicine, Daejeon St. Mary’s Hospital, College of Medicine, The Catholic University of Korea, Seoul 34943, Republic of Korea; 3 Department of Pathology, College of Medicine, Chungnam National University, Daejeon 34134, Republic of Korea; mkyeo83@gmail.com; 4 Thoracic and Cardiovascular Surgery, School of Medicine, Chungnam National University, Munhwa-ro 282, Jung-Gu, Daejeon 35015, Republic of Korea; dockang@cnu.ac.kr (M.-W.K.); yooychong@gmail.com (Y.C.); hansungjoon@hanmail.net (S.J.H.); 5 School of Life Science, Gwangju Institute of Science and Technology, Gwangju 61005, Republic of Korea; 6 Graduate School of Medical Science and Engineering, Korea Advanced Institute of Science and Technology (KAIST), Daejeon 34141, Republic of Korea; jp24@kaist.ac.kr (J.-E.P.); way2gokoh@kaist.ac.kr (Y.K.);; 7 Tomocube Inc., Daejeon 34141, Republic of Korea; jhlee@tomocube.com (J.L.); yk.park@kaist.ac.kr (Y.P.); 8 Department of Physics, Korea Advanced Institute of Science and Technology (KAIST), Daejeon 34141, Republic of Korea; 9 GENOME INSIGHT Inc., Daejeon 34051, Republic of Korea; 10 Central R&D Center, Medical & Bio Decision Co., Ltd., Suwon 16229, Republic of Korealeesh@mbdbiotech.com (S.-H.L.); goos4684@mbdbiotech.com (B.K.)

**Keywords:** cryobiopsy, lung cancer, organoid, high cancer cell purity, three-dimensional holotomography, drug screening

## Abstract

One major challenge associated with lung cancer organoids (LCOs) is their predominant derivation from surgical specimens of patients with early-stage lung cancer. However, patients with advanced lung cancer, who are in need of chemotherapy, often cannot undergo surgery. Therefore, there is an urgent need to successfully generate LCOs from biopsy specimens. Conventional lung biopsy techniques, such as transthoracic needle biopsy and forceps biopsy, only yield small amounts of lung tissue, resulting in a low success rate for culturing LCOs from biopsy samples. Furthermore, potential complications, like bleeding and pneumothorax, make it difficult to obtain sufficient tissue. Another critical issue is the overgrowth of normal lung cells in later passages of LCO culture, and the optimal culture conditions for LCOs are yet to be determined. To address these limitations, we attempted to create LCOs from cryobiopsy specimens obtained from patients with lung cancer (*n* = 113). Overall, the initial success rate of establishing LCOs from cryobiopsy samples was 40.7% (*n* = 46). Transbronchial cryobiopsy enables the retrieval of significantly larger amounts of lung tissue than bronchoscopic forceps biopsy. Additionally, cryobiopsy can be employed for peripheral lesions, and it is aided via radial endobronchial ultrasonography. This study significantly improved the success rate of LCO culture and demonstrated that the LCOs retained characteristics that resembled the primary tumors. Single-cell RNA sequencing confirmed high cancer cell purity in early passages of LCOs derived from patients with advanced lung cancer. Furthermore, the three-dimensional structure and intracellular components of LCOs were characterized using three-dimensional holotomography. Finally, drug screening was performed using a specialized micropillar culture system with cryobiopsy-derived LCOs. LCOs derived from cryobiopsy specimens offer a promising solution to the critical limitations of conventional LCOs. Cryobiopsy can be applied to patients with lung cancer at all stages, including those with peripheral lesions, and can provide sufficient cells for LCO generation. Therefore, we anticipate that cryobiopsy will serve as a breakthrough strategy for the clinical application of LCOs in all stages of lung cancer.

## 1. Introduction

Despite innovative drugs, such as targeted agents and immunotherapy, lung cancer is still the most common cause of death among patients with cancer [1,2]. Lung cancer is classified into non-small cell lung cancer (NSCLC) and small cell lung cancer (SCLC) according to its histological characteristics. In stages 1 and 2 of NSCLC, surgery can be considered; in stages 3 and 4, however, radiotherapy or chemotherapy is generally performed without surgery. In patients with SCLC, performing surgery is rare because micrometastasis and recurrence are common, even in the early stages [3,4].

Targeted therapy, immunotherapy, and antibody–drug conjugates have recently been developed for the treatment of lung cancer. The availability of these various treatment options makes it more important to select the optimal drug for each patient with lung cancer [5,6,7,8].

An organoid is a miniaturized and simplified version of an organ that is produced in a three-dimensional (3D) matrix and recapitulates the basic microanatomy and function of organs [9,10]. Studies developing cancer organoid models for personalized medicine and drug screening are in progress, and the culturing of lung cancer organoids (LCOs) is being vigorously executed [8,11,12,13,14,15]. However, most studies to date have made LCOs using surgical tissues, and published data have demonstrated that the success rate of culturing LCOs from biopsy samples is extremely low [12,16]. In addition, organoids made using surgical tissue have not yet resolved the critical issue that normal cells overgrow throughout passaging of LCOs [8,10,12,13,14,17]. Cancer cell purity is very low in the late passages of LCOs using surgical specimens [8,10,17]. To overcome these issues, Kim et al. [16] made minimal basal media for LCOs by depleting Wnt3a and Noggin. Several research groups have made LCOs with cancer cells from malignant effusion and metastatic sites [18,19]. However, the LCOs derived from these samples included only a small portion of patients with stage IV lung cancer.

There are two different methods of lung cancer biopsy: transthoracic needle biopsy (TTNB) for peripheral lesions and bronchoscopic biopsy for central lesions. Due to the thickness of the bronchoscope, peripheral lung lesions are very difficult to access via general bronchoscopy. TTNB sometimes causes severe complications, such as massive hemoptysis, pneumothorax, and hemothorax [20]. With the recent introduction of thin and ultrathin bronchoscopes and radial endobronchial ultrasound (EBUS), peripheral lesions can be approached, and tissues can be safely obtained [21]. Conventional forceps biopsy obtains only tiny pieces of tissue; thus, it is very difficult to obtain enough extra tissue to culture LCOs using this technique. More recently, transbronchial cryobiopsy has been applied for lung biopsy at several centers. The temperature of a cryoprobe drops to −100 °C in few seconds [22,23,24,25], and a large volume of adjacent tissues becomes attached to the cryoprobe. Chunks of tissue can, thus, be obtained via cryobiopsy, providing enough specimens for pathologic diagnosis and other experiments, such as development of LCOs.

We were able to safely obtain much more lung cancer tissue using cryobiopsy than using TTNB or forceps biopsy. LCOs and normal lung organoids were successfully cultured using the cryobiopsy specimens. Additionally, we optimized the process of tissue digestion. We then validated the LCOs via many methodologies, including immuno-histochemistry (IHC), holotomography, and single-cell RNA sequencing (scRNA-seq). Finally, we performed a drug sensitivity test using cryobiopsy specimen-derived LCOs for clinical application.

## 2. Materials and Methods

### 2.1. Radial EBUS and Transbronchial Lung Cryobiopsy

All procedures were performed under moderate-to-deep sedation using midazolam, fentanyl, and/or propofol without general anesthesia. After sedation, a bronchoscope was introduced orally (or through an endobronchial tube in patients who were intubated), and 1% lidocaine hydrochloride solution was administered through the working channel for local anesthesia. The decision whether to use radial EBUS or a guide sheath (GS) was determined according to the location of the lesion and the type of bronchoscope. For directly observed central lesions, a 1.8 mm forceps biopsy was performed, followed by cryobiopsy through the working channel. For peripheral pulmonary lesions, radial EBUS and a GS were used to approach the target lesion, and a 1.5 mm forceps biopsy was then performed, followed by cryobiopsy using a 1.1 mm cryoprobe through the GS [26].

We located the target lesions with radial EBUS and obtained lung tissues with biopsy forceps more than three times. Next, we obtained lung tissues via cryobiopsy. Cryobiopsy yielded spherical specimens that surrounded the metal tip; the amount of tissue esd usually more than 10 times than that obtained via a forceps biopsy specimen (standard oval forceps, FB-231D; Olympus, Shinjuku Ward, Japan) when using a 1.9 mm cryoprobe (CRYO2; Erbe Elektromedizin GmbH, Tübingen, Germany) [24].

### 2.2. Cell Isolation

Lung cancer tissues stored in DMEM/F12 media (Gibco, Waltham, MA, USA) that contained 1% penicillin and streptomycin (Gibco) were transported to the laboratory and processed within 12 h of removal from the patients. Upon arrival, the lung cancer tissues were photographed, and their volumes were measured.

For mechanical dissociation, the tissues were cut to as small a size as possible using surgical scissors, processed with a handheld microtube homogenizer on ice, and then resuspended in DMEM/F12 that contained 1% penicillin and streptomycin. The suspension was filtered through a 70 µm strainer (SPL Life Sciences, Pocheon-si, Republic of Korea). After the membranes were rinsed, the residual impurities on the membranes were discarded together with the strainer. The strained cells were centrifuged at 500× *g* for 5 min at 4 °C, and the supernatant was discarded. The pellet was incubated with red blood cell lysis buffer (BioLegend, San Diego, CA, USA) for 10 min at room temperature. Cold Dulbecco’s phosphate-buffered saline (DPBS) containing 10% fetal bovine serum (FBS) (Welgene, Nancheon, Republic of Korea) was added, and centrifugation was performed at 500× *g* for 5 min at 4 °C. The cell pellet was washed once with cold DPBS and centrifuged again for enzyme digestion. The organoid culture media contained fibroblast growth factors 7 and 10, noggin, R-spondin, and other components.

### 2.3. Culture and Expansion of Organoids

To culture LCOs in a multi-well plate, the LCOs were resuspended in cold-reduced growth factor basement membrane extract type 2 (BME) (R&D Systems, Minneapolis, MN, USA). The seeding density was then adjusted to approximately 1000 single cells per 20 µL drop of the BME suspension into a 48-well plate (SPL Life Sciences, Pocheon-si, Republic of Korea) and allowed to solidify at 37 °C for 10 min. After gelation, 120 µL LCO culture medium was added to the well, and the plate was transferred to an organoid culture incubator at 37 °C with 5% carbon dioxide. The composition of the LCO culture medium is shown in Supplementary Table S1. The medium was refreshed weekly.

### 2.4. Passaging of Organoids

For passage, organoids (2–3 weeks after starting the culture) were harvested from cold DPBS (Welgene) on the plate and centrifuged at 500× *g* for 5 min at 4 °C. The supernatant was discarded, and the BME was digested with 200 µL TrypLE Express (Gibco) for approximately 10 min at 37 °C. The trypsinization was stopped by the addition of 1 mL cold DPBS containing 10% FBS (Welgene). The organoids were centrifuged at 500× *g* for 5 min at 4 °C, washed once with cold DPBS, and centrifuged again. The pellets were resuspended in cold BME to allow the formation of new organoids.

### 2.5. Cryopreservation of Organoids

Organoids that were cultivated during passages with sufficient quantities and a diameter reaching 100 to 150 µm were banked. To stock the organoids, they were harvested using cold DPBS (Welgene) and then centrifuged at 500× *g* for 5 min at 4 °C. The supernatant was discarded, and the BME was digested with 200 µL TrypLE Express (Gibco) for approximately 10 min at 37 °C. The trypsinization was stopped by the addition of 1 mL cold DPBS containing 10% FBS (Welgene). The organoids were centrifuged at 500× *g* for 5 min at 4 °C, washed once with cold DPBS, and centrifuged again. The supernatant was removed, and the organoid pellet was resuspended in Recovery Cell Culture Freezing Medium (Gibco). The stock vials were stored in a freezing container at −80 °C. The following day, the vials were transferred to a liquid nitrogen tank.

### 2.6. Hematoxylin and Eosin Staining and IHC

Tissues and organoids were fixed in 4% paraformaldehyde, followed by dehydration, paraffin embedding, sectioning, and standard hematoxylin and eosin (H&E) staining and IHC staining. Next, the samples were incubated with primary antibodies, including anti-thyroid transcription factor-1 (TTF-1) (1:200 dilution; Invitrogen Signaling Technology), anti-p40 (1:200 dilution; Cell Signaling Technology), anti-cytokeratin 5 and 6 (1:200 dilution, #MA5-12429; Invitrogen), and anti-programmed death ligand-1 (anti-PD-L1) (1:200 dilution, #13684; Cell Signaling Technology). The sections were subsequently incubated with secondary antibodies (#AI-2000 and #AI-1000; Vector Laboratories, Newark, CA, USA) at 1:5000 dilution and visualized using an ultraView Universal DAB Detection kit (Ventana Medical Systems, Oro Valley, AZ, USA). Nuclei were counterstained with Harris hematoxylin. Images were acquired via a Leica Eclipse E600 microscope [16].

### 2.7. Label-Free 3D Imaging of Organoids via Holotomography

The organoids were harvested from cold DPBS (Welgene) from the plate and centrifuged at 500× *g* for 5 min at 4 °C. The supernatant was discarded, washed once with cold DPBS, and centrifuged again. This step was repeated three times to dissolve the BME surrounding the organoids. The organoid pellets were fixed in 4% paraformaldehyde (Biosesang) for 24 h at 4 °C, centrifuged at 500× *g* for 5 min at 4 °C, washed once with DPBS, and centrifuged again. This step was repeated two times. The organoid pellets in DPBS were transferred to a 50 mm imaging dish with a #1.5 bottom coverslip (TomoDish; Tomocube). Three-dimensional refractive index tomograms with a Z-scan range of 140 μm were acquired using HT-X1 holotomography (Tomocube) and visualized via image analysis software (TomoAnalysis version 1.5; Tomocube).

### 2.8. scRNA-seq

Cryopreserved initial-passage organoid cell stocks were thawed in a 37 °C water bath for 1 min and transferred to DMEM/F-12 (Gibco), followed by centrifugation at 500× *g* for 5 min at 4 °C. The cells were further washed twice with cold DPBS (Gibco) containing 0.04% BSA (Miltenyi Biotec) and filtered through 40 µm cell strainers (Flowmi). Cell suspensions from two different donors were pooled to equal cell concentrations. Next, the pooled cell suspensions were loaded, targeting recovery of 10,000 cells per channel to construct scRNA-seq libraries using a Chromium Next GEM Single Cell 5′ kit v2 (10× Genomics) according to the manufacturer’s instructions. The generated libraries were sequenced on an Illumina NovaSeq 6000 at a paired-end 150 bp reads configuration, which aimed to generate 400 million reads per library.

The reads were aligned to the GRCh38 human genome (2020-A; 10× Genomics) using Cell Ranger (v6.0.2; 10× Genomics) with an include-introns parameter. The donors were demultiplexed using souporcell (v2.0) [27] to two clusters based on common variants. Ambient RNA counts were eliminated via CellBender (v0.2.1).

Filtered count matrices were further processed with Scanpy (v1.9.1) [28]. Low-quality cells with <2000 total counts, <500 genes, >15% mitochondrial gene counts, and >0.4 Scrublet (v0.2.3) [29] doublet detection scores were filtered out, and only souporcell singlets were retained. Raw counts were normalized per cell to 10,000 and ln(x + 1) transformed for further use as gene expression values.

The data were split into two donors based on souporcell assignment for downstream analyses. The top 2000 highly variable genes were selected using the scanpy.pp.highly_variable_genes function and the seurat_v3 method [30] for each datum. After data scaling to unit variance, principal component analysis of the selected highly variable genes was performed. A neighborhood graph was built from the top 50 principal components and visualized using uniform manifold approximation and projection (UMAP).

Clustering was performed using the Leiden method [31] at 2.0 resolution. Low-quality and stressed clusters displaying low total counts, low numbers of genes, and high mitochondrial gene counts were discarded, and the remaining data were reprocessed from the highly variable gene selection step. Cell cycle scores were calculated with the scanpy.tl.score_genes_cell_cycle function. Each cluster was manually annotated by inspecting marker genes.

Copy number variation (CNV) was inferred using infercnvpy (v0.1.0) with a 100 running window size. Pre-annotated normal cell types were provided as a reference.

### 2.9. Drug Sensitivity Test

Organoids were harvested and mixed with high-concentration growth factor reduced Matrigel (basement membrane extracted from Engelbreth–Holm–Swarm mouse sarcoma; Corning Life Sciences Catalog #354263, USA) to a final cell concentration of 5 × 105 cells/mL for the pillar plate. Next, matrigel–cell mixtures (1.5 µL) were spotted using a pre-cooled ASFA^®^ spotter (Medical & Bio Decision, Suwon, Republic of Korea) onto coated pillar plates, which were then incubated face down for 15 min at 4 °C to allow cell aggregation at the bottom of the spots. Next, the plates were placed in a humidity chamber at 37 °C to allow spot gelation. The pillar plates were subsequently combined with well plates containing fresh medium. After 3 days, the cells were treated with six concentrations of EGFR- or ALK-targeted agents using the ASFA^®^ spotter. Cell viability was determined via staining with 0.5 µM calcein-AM for 1 h at 37 °C in a humified incubator and washing twice with PBS for 20 min. The organoid spots were scanned with an automatic optical fluorescence scanner (ASFA^®^ scanner; Medical and Bio Decision, Republic of Korea). The microscope in the scanner automatically focused on the cell spots by moving in the z-direction, and it took 384 individual pictures from a single stained pillar/well plate at 4× magnification. Next, the 384 pictures of the cell spots were consolidated into a single JPEG image for data analysis. The scanned images were analyzed using CellAnalyzer version 2.0 (Medical and Bio Decision). After scanning the cells, the organoid spots were transferred to a new 384-well plate containing CellTiter-Glo^®^ 3D reagent to measure the intracellular ATP levels via luminescence activity.

## 3. Results

### 3.1. High Success Rate of LCO Culture Derived from Cryobiopsy Samples

**Figure 1:**
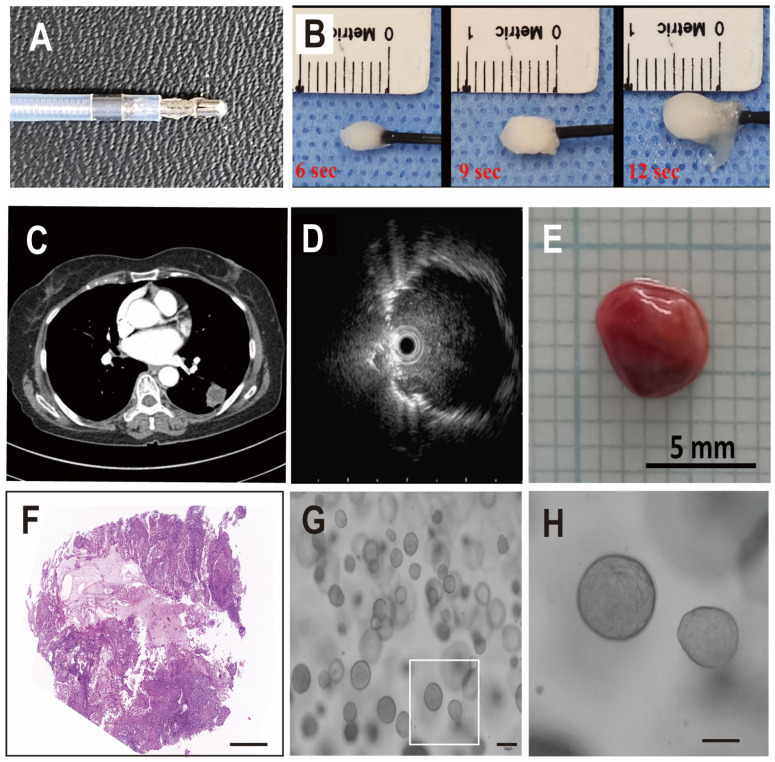
Transbronchial cryobiopsy for all stages of lung cancer. (**A**) Photograph of a cryobiopsy probe. (**B**) Demonstrative photographs that show the concept of cryobiopsy. (**C**,**D**) Representative images of peripheral lung cancer lesions and radial EBUS findings. (**E**) Typical cryobiopsy specimen of lung cancer. (**F**) Representative H&E staining of cryobiopsy-derived lung cancer. (**G**,**H**) Representative images of LCOs (scale bar: 100 µm).

Cryobiopsy can freeze adjacent lesions within a few seconds (Figure 1A,B). We approached peripheral lung cancer lesions with ultrathin bronchoscopy and checked the accuracy of the probe location with radial EBUS (Figure 1C,D). When tissue was obtained using the cryobiopsy probe, the tissue volume was more than 10 times larger than that of conventional forceps biopsy samples (Figure 1E). H&E staining of the cryobiopsy samples showed high cancer cell purity, suggesting that cryobiopsy of the tumor core guarantees high purity of cancer cells in LCOs (Figure 1F). Generally, the forceps biopsy specimens contained about 1 to 5 × 10^5^ cells, and the cryobiopsy specimens contained more than 1 to 5 × 10^6^ cells. We tried to make LCOs using the specimens derived from cryobiopsy (*n* = 113) (Figure 1G,H). The prevalence of adenocarcinoma accounted for 50.4% (*n* = 57), squamous cell cancer accounted for 24.8% (*n* = 28), SCLC accounted for 4.4% (*n* = 5), and undetermined types accounted for 20% (*n* = 23). In terms of stages, 29.2% (*n* = 33) of patients had stage I lung cancer, 10.6% (*n* = 12) had stage II lung cancer, 11.5% (*n* = 13) had stage III lung cancer, 23% (*n* = 26) had stage IV lung cancer and 25.6% (*n* = 29) had an undetermined stage of lung cancer. Some patients were unable to complete certain additional tests, such as IHC for tissue analysis, Magnetic Resonance Imaging for the detection of brain metastases, and Positron Emission Tomography. This issue resulted in incomplete pathological confirmation and staging, either due to their transfer or other circumstances.

Overall, the initial success rate of establishing LCOs from the cryobiopsy samples was 40.7% (*n* = 46). The success rate of initial culture is critically important for the clinical application of LCOs in personalized medicine. In this study, we generally preserved the organoid samples for future utilization after two or three successful passages. We also confirmed that some organoids continued to grow up to 8 to 10 passages and found that most of the stocked organoids regrew after thawing.

### 3.2. Cryobiopsy Tissue-Derived LCOs Recapitulate the Pathology and 3D Structure of Primary Cancer

**Figure 2:**
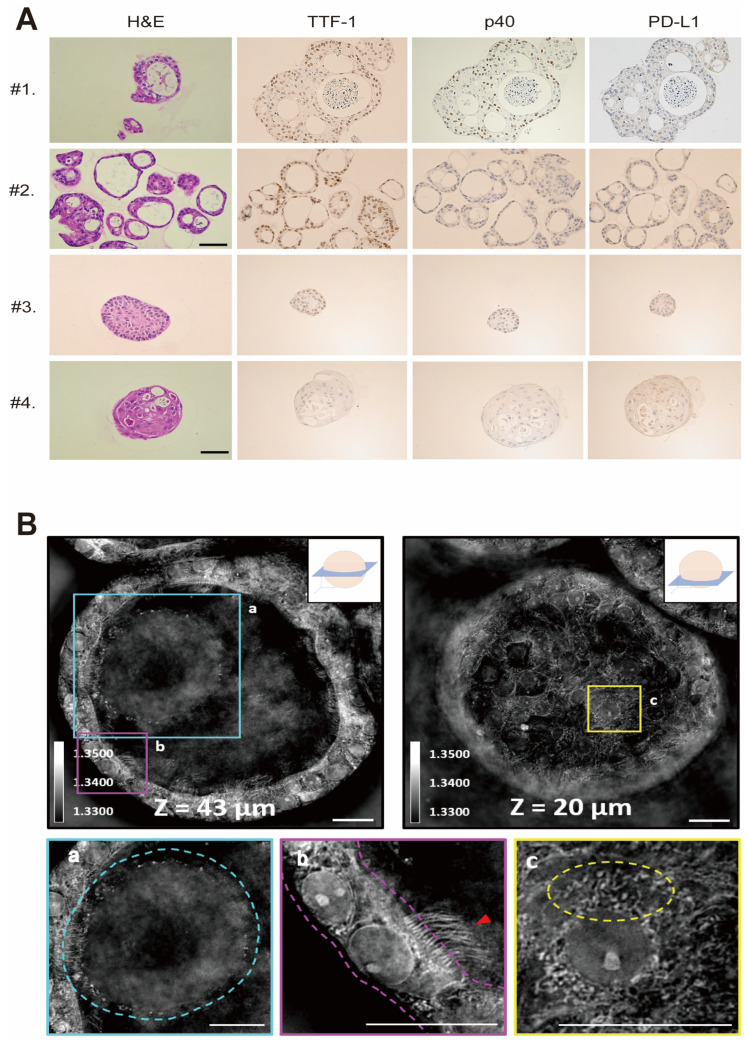
Morphology of normal lung organoid and LCO from cryobiopsy specimens. (**A**) Representative images of H&E staining and IHC staining of LCOs. Patients #1 and #2 were diagnosed with NSCLC, and Patients #3 and #4 were diagnosed with SCLC. The profiles of IHC, including TTF-1, p40, and PD-L1, were correlated with the primary tumors. (**B**) Holotomography of LCOs taken in multiple Z sections. The images demonstrate (**a**) mucosal layer, (**b**) apicobasal polarization, ciliated cells (red arrowed) and (**c**) various intracellular organelles, such as mitochondria and lipid droplets.

We made a paraffin section of an LCO and then performed H&E staining. The general morphology of the LCO showed a pathologic phenotype similar to that of the primary lung cancer tissue (Figure 2A). IHC also demonstrated that the LCO expressed similar expression patterns of significant markers, including TTF-1, p40, and PD-L1. TTF-1 is a marker of lung adenocarcinoma, and p63 is an indicator of squamous cell cancer. PD-L1 is related to immune check point inhibitor, and the expression level is predictive biomarker for immunotherapy. These data suggest that LCOs derived from cryobiopsy specimens recapitulate the basic characteristics of primary lung cancer tissues.

To evaluate the 3D structure and subcellular characteristics of LCOs with a non-invasive imaging technique, we used a low-coherence holotomography imaging system. (Figure 2B). Low-coherence holotomography is suitable for examining the morphological features of 3D multicellular specimens without any pre-treatment, such as staining or fluorescence marker protein expression [32]. Refractive index tomograms obtained through holotomography provide not only label-free optical sectioning [5,23], but also information on the dry mass of subcellular compartments. Unlike spheroids, which are composed of only one type of cell, organoids are composed of several types of cells. In the present study, the refractive index tomograms showed the defined structures of LCOs in three dimensions, the lumen structure in the center of the organoid, and the surrounding cells. These data confirmed that LCOs are composed of several kinds of cells. The secreted mucus in the lumen was distinguished by a slightly higher refractive index distribution than that in the airway. Apicobasal polarization was observed in the cells surrounding the cleared lumen, and ciliated cells were clearly identified based on their multiciliate structures. The subcellular structures of each cell were also observed, such as nuclei, nucleoli, mitochondria, and lipid droplets. These images confirmed that the grown organoids recapitulated the structure and function of the human lung in vivo (Figure 2B).

With the utilization of high-resolution imaging technology, it becomes feasible to analyze the variations in LCO morphology based on lung cancer type and the development of resistance to anticancer drugs. Furthermore, the challenge of distinguishing between normal lung organoids and LCOs, which has been a significant issue, can be resolved by employing 3D live image analyses.

### 3.3. High Cancer Cell Purity of Cryobiopsy Tissue-Derived LCOs

**Figure 3:**
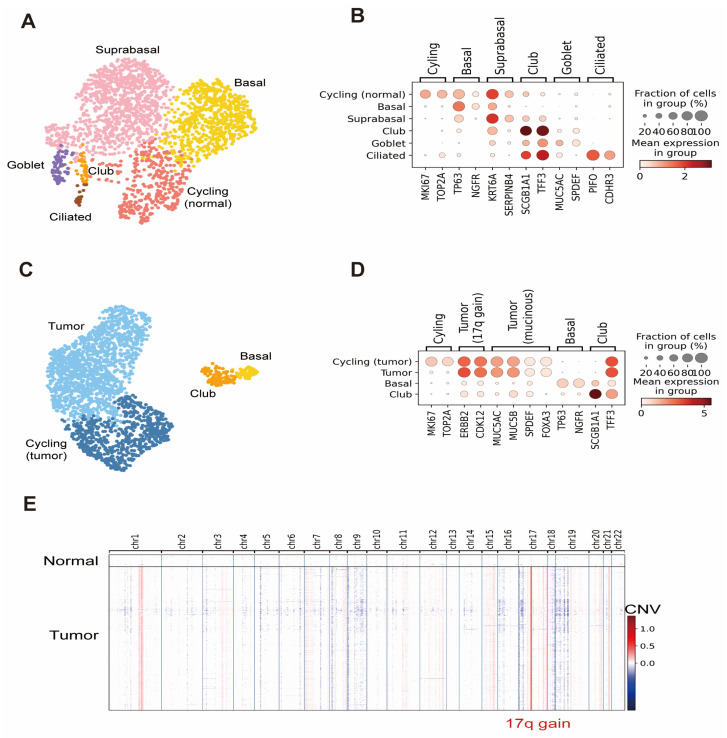
Results of scRNA-seq analyses of lung organoids and LCOs. (**A**) UMAP of normal lung organoid cell types and states. (**B**) Dot plot with expression of selected marker genes in a normal lung organoid. (**C**) UMAP of LCO cell types and states. (**D**) Dot plot with expression of selected marker genes in an LCO. (**E**) Heatmap of inferred CNV values per chromosome for each cell in the LCO data. A distinct chromosome 17q gain in tumor cells is marked.

Normal lung organoids were cultured using samples confirmed to contain normal lung epithelial cells without cancer cells. Next, we performed scRNA-seq on normal lung organoids and LCOs that we established using cryobiopsy specimens. We obtained 2138 normal lung organoid cells and 2080 LCO cells after removing low-quality and stressed cells (Figure 3A). Cell type annotation based on marker gene expression [9] showed that the lung organoids contained diverse epithelial cell types and states in the human lung, including cycling cells (MKI67, TOP2A), basal cells (TP63, NGFR), suprabasal cells (KRT6A, SERPINB4), club cells (SCGB1A1, TFF3), goblet cells (MUC5AC, SPDEF), ciliated cells (PIFO, CDHR3), and tumor cells (Figure 3B–D). Although rare epithelial cell types could not be annotated because of the low number of profiled cells, the expression of marker genes for rare cell types, including deuterosomal cells (FOXN4, DEUP1), neuroendocrine cells (GRP, ASCL1), tuft cells (POU2F3, ASCL2), and ionocytes (FOXI1, ASCL3), was also detected in normal lung organoid data (Figure 3E).

Tumor cell annotation was confirmed via CNV inference. We identified 7.69% (160 of 2080) of normal cells in the LCO data. The purity of cancer cells in the late passage remains to be checked in multiple organoids.

CNV inference further revealed that the tumor cells contained the chromosome 17q gain signature, which is associated with amplification of the ERBB2 oncogene [33]. This signature, together with high expression of ERBB2 and co-amplified CDK12 in organoids, recapitulated the genomic changes from the patient’s original tumor [34]. Additionally, markers of invasive mucinous adenocarcinoma (MUC5AC, MUC5B, SPDEF, FOXA3) [12] were highly expressed in organoids, retaining the histological characteristics of the patient’s original tumor.

Raw sequencing data and processed AnnData objects have been deposited in the Gene Expression Omnibous (GEO) with accession number GSE230537 and will be publicly available as of the date of publication.

These data suggest that LCOs made from cryobiopsy specimens obtained from the core of lung cancer exhibit high cancer cell purity in the early passages. For advanced and metastatic lung cancer patients, these early passage LCOs with a high degree of cancer cell purity can be employed for drug screening to determine the most effective medication for treating patients with lung cancer.

### 3.4. Drug Screening Based on Cryobiopsy Tissue-Derived LCOs for Precision Medicine

**Figure 4:**
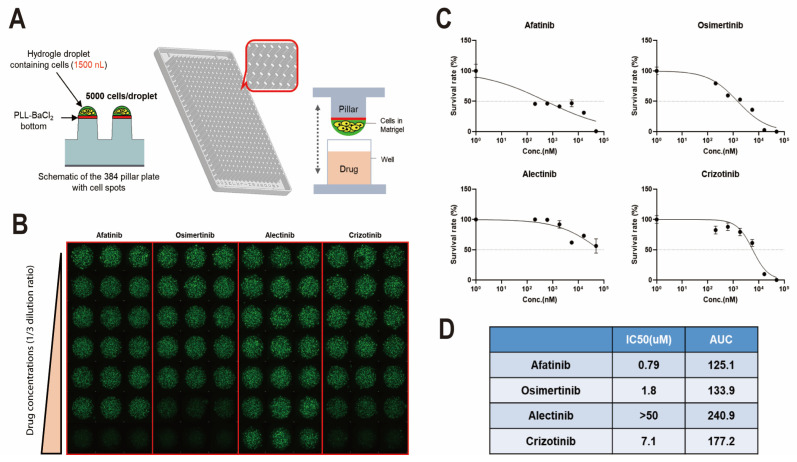
Drug sensitivity test using cryobiopsy specimen-derived LCOs for precision medicine. (**A**) LCOs were seeded in a micropillar with 5000 cells/pillar and soaked in medium. (**B**) After treatment with multiple doses of several drugs, the viability of LCOs was checked using the fluorescent dye calcein AM. The maximum drug concentration was 20 µM (each drug). (**C**) Survival rates of LCOs after multiple doses of afatinib, osimertinib, alectinib, and crizotinib. (**D**) The IC50 and AUC of each drug were calculated.

Finally, we performed a drug sensitivity test with LCOs for future precision medicine. We utilized LCOs derived from lung cancer that exhibited EGFR mutation. In total, 5000 single cells per pillar were seeded in 1.5 µL medium and incubated for 3 days (Figure 4A). Next, they were treated with multiple doses of several EGFR-TKI and ALK inhibitors for another 3 days to check the half-maximal inhibitory concentration (IC50) and area under the curve (AUC). On the sixth day, the viability of the LCOs was checked using the fluorescent dye Calcein AM, and intracellular ATP levels were checked using a CellTiter-Glo 3D cell viability assay (G9681; Promega) (Figure 4B,C). The results demonstrated that LCOs from patients with EGFR-mutated lung cancer were sensitive to EGFR-TKI but not to ALK inhibitors (Figure 4D). This result suggests that LCOs from cryobiopsy specimens can be used for drug screening and personalized medicine.

## 4. Discussion

Treatment of lung cancer has been developing rapidly, and many effective treatments, such as targeted therapy, immunotherapy, and antibody–drug conjugates, can be used in a clinical setting [1,35,36]. Next-generation sequencing is a very popular method of identifying optimal drugs for patients with lung cancer. Although it provides genetic information, it cannot predict the exact responsiveness to chemotherapies such as immunotherapy [37]. In addition, there is great variation in the response to treatment depending on patients’ individual characteristics, even when they have the same driver mutation. Therefore, there is still an unmet need for biomarkers to predict treatment responses more precisely.

LCOs can be utilized for personalized medicine and high-throughput drug screening. When co-cultured with lymphocytes, fibroblasts, and endocytes, LCOs can mimic the tumor microenvironment. Therefore, they have the potential to predict the response to immunotherapy, in addition to cytotoxic chemotherapy and targeted therapy. Several studies have shown that LCOs recapitulate the primary lung cancer and predict the drug response. However, there are still several critical limitations of LCOs for lung cancer therapy. Firstly, until now, almost all LCOs were derived from surgical specimens of early lung cancer patients who usually do not need urgent chemotherapy. Secondly, concern exists regarding low cancer cell purity in late-passage LCOs derived from surgical specimens. Many studies have revealed that normal lung cells overgrow and that there are few cancer cells in late-passage LCOs [8,12,14,17,38]. In a previous study, Nutlin 3a, which is an MDM2 inhibitor, was utilized to induce apoptosis of p53 wild-type cells; however, 30–40% of lung cancer cases have wild-type p53. The main reasons for overgrowth of normal lung cells in late passages is that significant numbers of normal lung cells are present in surgically resected lung [39] and culture media that contain fibroblast growth factors 7 and 10, noggin, and SB4 can promote the proliferation of normal lung cells [16]. Finally, the success rate of culturing LCOs from small biopsy specimens is very low. It is difficult to obtain adequate tissue samples via conventional forceps biopsy for central lesions and via TTNB for peripheral lesions [21,22,40]. Lung biopsy is relatively invasive and has a high risk of severe complications, such as massive hemoptysis and pneumothorax [40]. In order to enhance the success rate of primary cancer cell culture, the utilization of conditional reprogramming has been explored. By employing a co-culture approach involving fibroblast cells and the supplementation of Y-27632, the success rate has been significantly improved [41]. Further optimization of the conditional reprogramming technique holds the potential to further enhance the success rate of LCO cell culture.

To overcome these limitations, our group has utilized cryobiopsy-derived specimens for LCO culture for the first time. Cryobiopsy was recently introduced in lung biopsy to diagnose interstitial lung disease or lung cancer [22,23,42,43]. With cryobiopsy, we obtained even larger lung cancer tissues from patients with all stages of lung cancer and both central and peripheral lesions. Cryobiopsy from the core of lung cancer lesions showed very high numbers of cancer cells, which would guarantee high purity of lung cancer cells, even in late passages. More optimized media for LCOs would be helpful to maintain the high purity of cancer cells.

## 5. Conclusions

For personalized medicine of advanced NSCLC and all stages of SCLC, which require chemotherapy, LCOs must be made using biopsy tissue, rather than surgical tissue. Until now, most LCOs have been made from surgical tissues of early lung cancer patients, and the overgrowth of normal cells in such LCOs has been the biggest limitation of LCOs. The success rate of LCO culture using biopsy tissue has been very low, and one of the main reasons was the lack of tissue samples. To overcome the limitations of LCO culture, we created LCOs with cryobiopsy specimens. Tissues obtained via cryobiopsy from the core of late-stage lung cancer lesions were mostly composed of cancer cells. The cryobiopsy specimens contained 5 to 10 times more cells than the conventional forcep biopsy specimen. The initial success rate of establishing LCOs from the cryobiopsy samples was over 40%. We conclude that cryobiopsy is a breakthrough method for creating LCOs for practical clinical applications because it can be performed on most lung cancer patients and provide significantly larger tissue specimens with high purity of cancer cells.

## Data Availability

The datasets used and/or analyzed during the current study are available from the corresponding author on request.

## Supplementary Materials

The following supporting information can be downloaded at: https://www.mdpi.com/article/10.3390/cells12141854/s1, Table S1: Human Lung airway cell organoid culture media ([10,16]).
