# Supplementary material for: Cryobiopsy: A Breakthrough Strategy for Clinical Utilization of Lung Cancer Organoids
Source: Cells. 2023 Jul 14;12(14):1854. doi: 10.3390/cells12141854 (PMC10377875; doi:10.3390/cells12141854)
Supplement: Supplementary file 1 [file cells-12-01854-s001.zip › cells-2416213-supplementary.pdf]

Supplementary data

Table S1.

**Human Lung airway cell organoid culture media ([10,16])**		
	**Stock concentration**	**Final concentration**
**AdDMEM/F12**		
**HEPES**	1M	10mM
**Glutamax**	100X	1X
**P/S**	100X	1X
**R-spondin1**	500ug/ml	500ng/ml
**hFGF7**	10ug/ml	25ng/ml
**hNoggin**	100ug/ml	100ng/ml
**hFGF10**	100ug/ml	100ng/ml
**B27 supplement**	50X	1X
**N-acetylcysteine**	500mM	1.25mM
**Primocin**	50mg/ml	50ug/ml
**SB431542**	10mM	500nM
**Nicotinamide**	1M	5mM
**A83-01**	1mM	500uM
**Y27632**	10mM	5uM
